# Supplementary material for: Gene therapy for progressive familial intrahepatic cholestasis type 3 in a clinically relevant mouse model
Source: Nat Commun. 2019 Dec 13;10:5694. doi: 10.1038/s41467-019-13614-3 (PMC6910969; doi:10.1038/s41467-019-13614-3)
Supplement: Supplementary file 1 — Supplementary Information [file 41467_2019_13614_MOESM1_ESM.pdf]

Supplementary Information

Gene therapy for progressive familial intrahepatic cholestasis type 3 in a clinically relevant mouse model

Weber, et al.

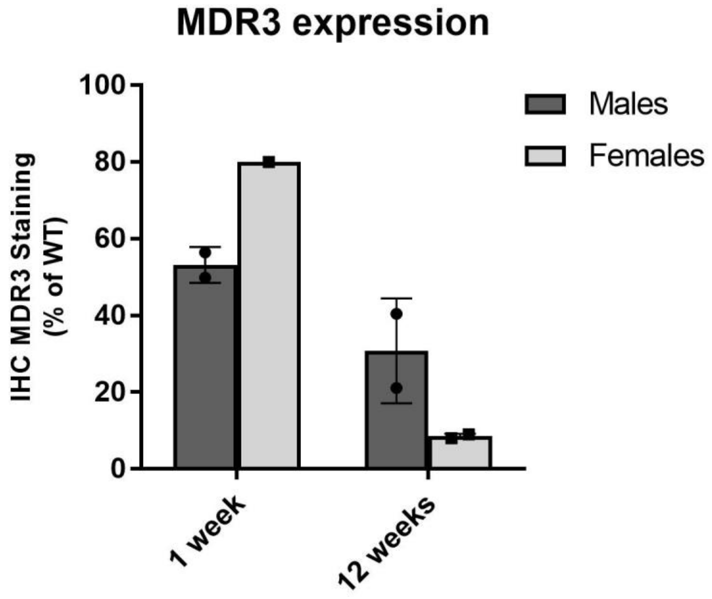

Supplementary Figure 1. Quantification of AAV-MDR3-Aco expression in *Abcb4*$^{-/-}$ mice. Two-week-old *Abcb4*$^{-/-}$ mice were treated 1X with 1x10^{14} VG/kg of AAV-MDR3-Aco and protein expression was detected by IHC one or twelve weeks later and quantified as the percent area of tissue that stained positive for MDR3 expression and normalized to levels in WT mice. (1 week: n=2M/1F; 12 weeks: n=2M/2F). Statistics: unpaired t test (males): not significant (t=2.194 df=2) Source data are provided as a Source Data file.

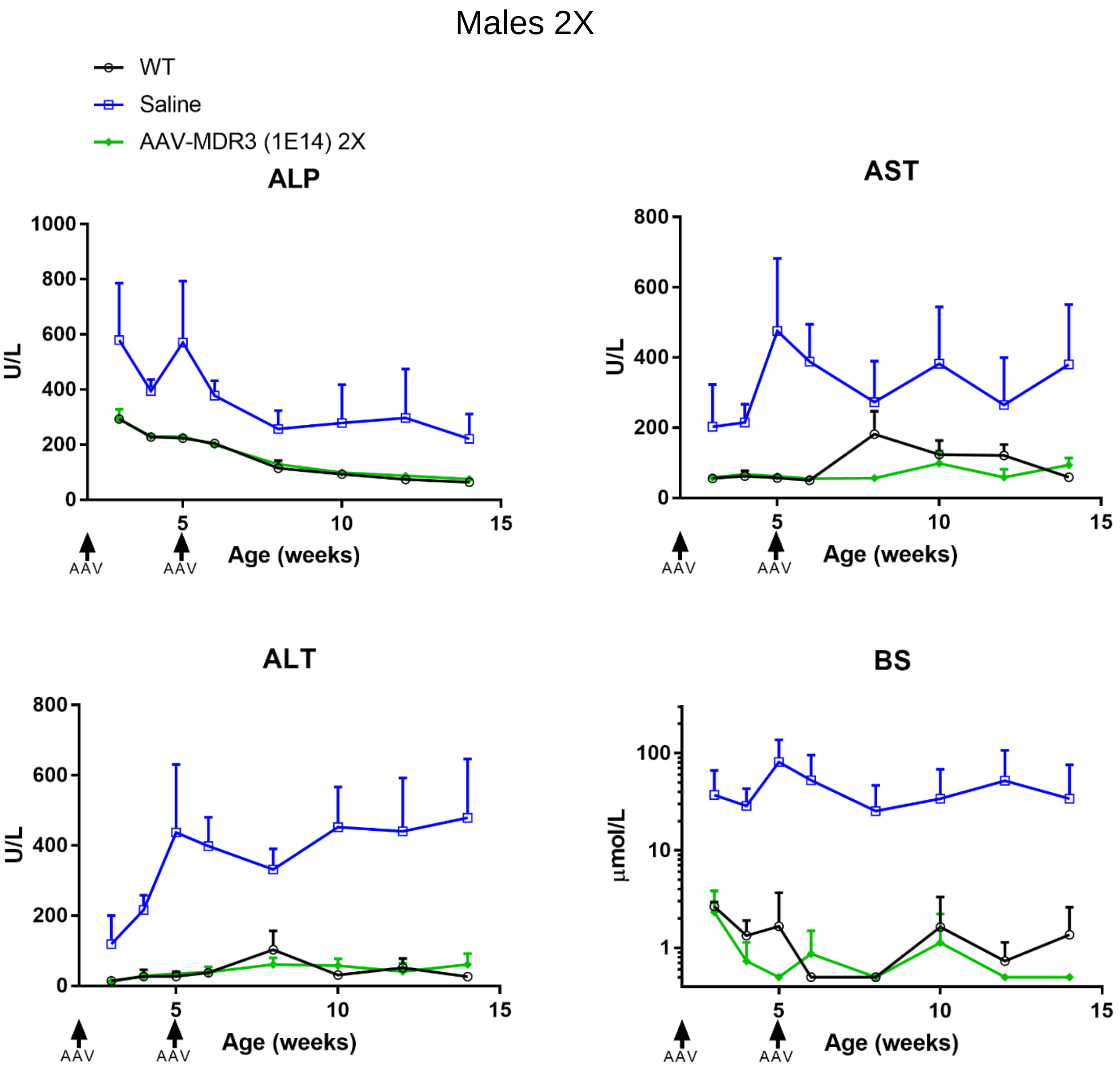

Supplementary Figure 2. Serum biomarker levels for AAV-MDR3-Aco-treated *Abcb4*$^{-/-}$ male mice through twelve weeks. The therapeutic effect of AAV treatment is shown in males that were treated with AAV-MDR3-Aco at 1x10^{14} VG/kg two times (green, filled diamonds; n=3M) using as controls saline-treated (blue, open squares; n=4M) and untreated *Abcb4*$^{+/+}$ (WT) mice (black, open circles; n=3M). Data are presented as mean + SD. ALP, alkaline phosphatase; ALT, alanine transaminase; AST, aspartate transaminase; BS, bile salts. Source data are provided as a Source Data file.

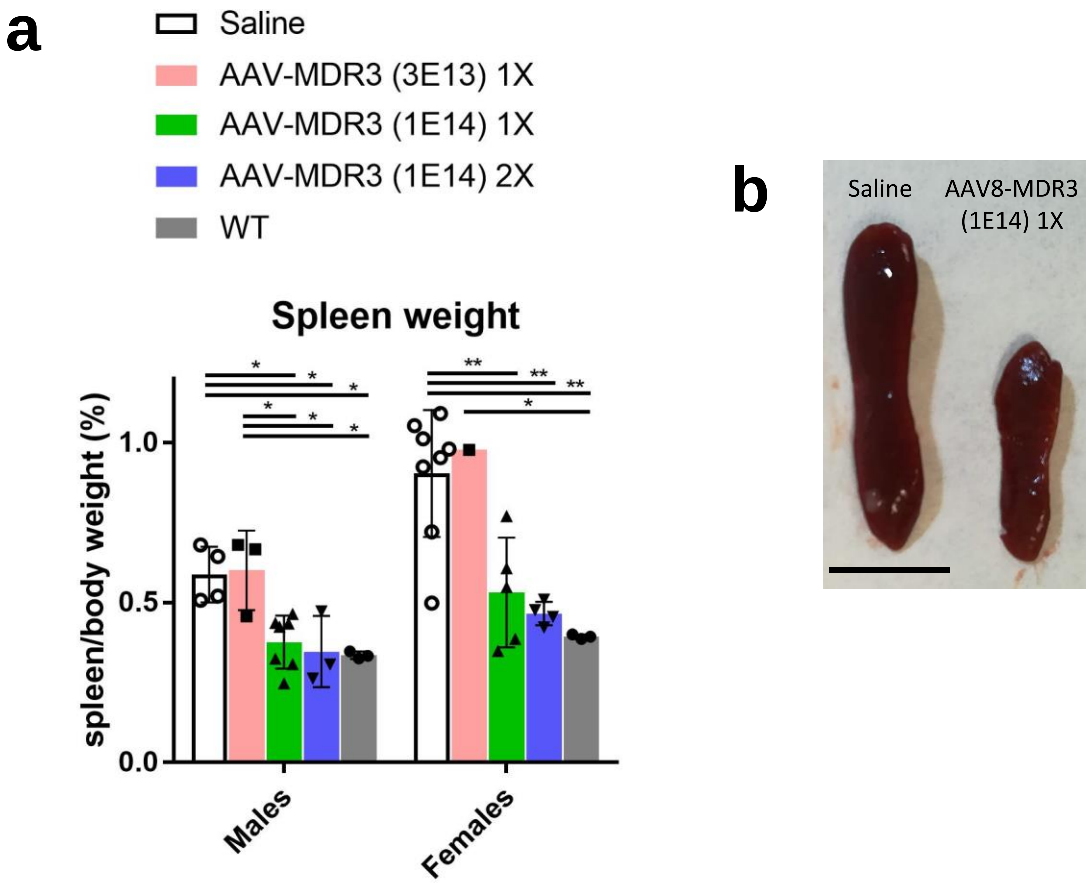

Supplementary Figure 3. Spleen weight for *Abcb4*$^{-/-}$ mice treated with AAV-MDR3-Aco. At time of sacrifice, spleen weight as a percent of body weight (**a**) was measured for *Abcb4*$^{-/-}$ mice treated with AAV-MDR3-Aco at 3×10^{13} VG/kg 1X (pink bars; n=3M/1F), at 1×10^{14} VG/kg 1X (green bars; n=7M/5F), or at 1×10^{14} VG/kg 2X (blue bars; n=3M/4F), as well as saline-treated controls (white bars; n=4M/8F) and untreated WT mice (grey bars; n=3M/3F). Statistics (one-way ANOVA/Tukey's multiple comparisons test): *, $p<0.05$; **, $p<0.01$; data are presented as mean ± SD; F values and degrees of freedom (numerator, denominator): (**a**) males: $F(4,15) = 7.864$, females: $F(4,16) = 10.31$; (**b**) Images of representative spleens from females treated with Saline (left) or AAV-MDR3-Aco at 1×10^{14} VG/kg 1X (right). Scale bar = 1 cm. Source data are provided as a Source Data file.

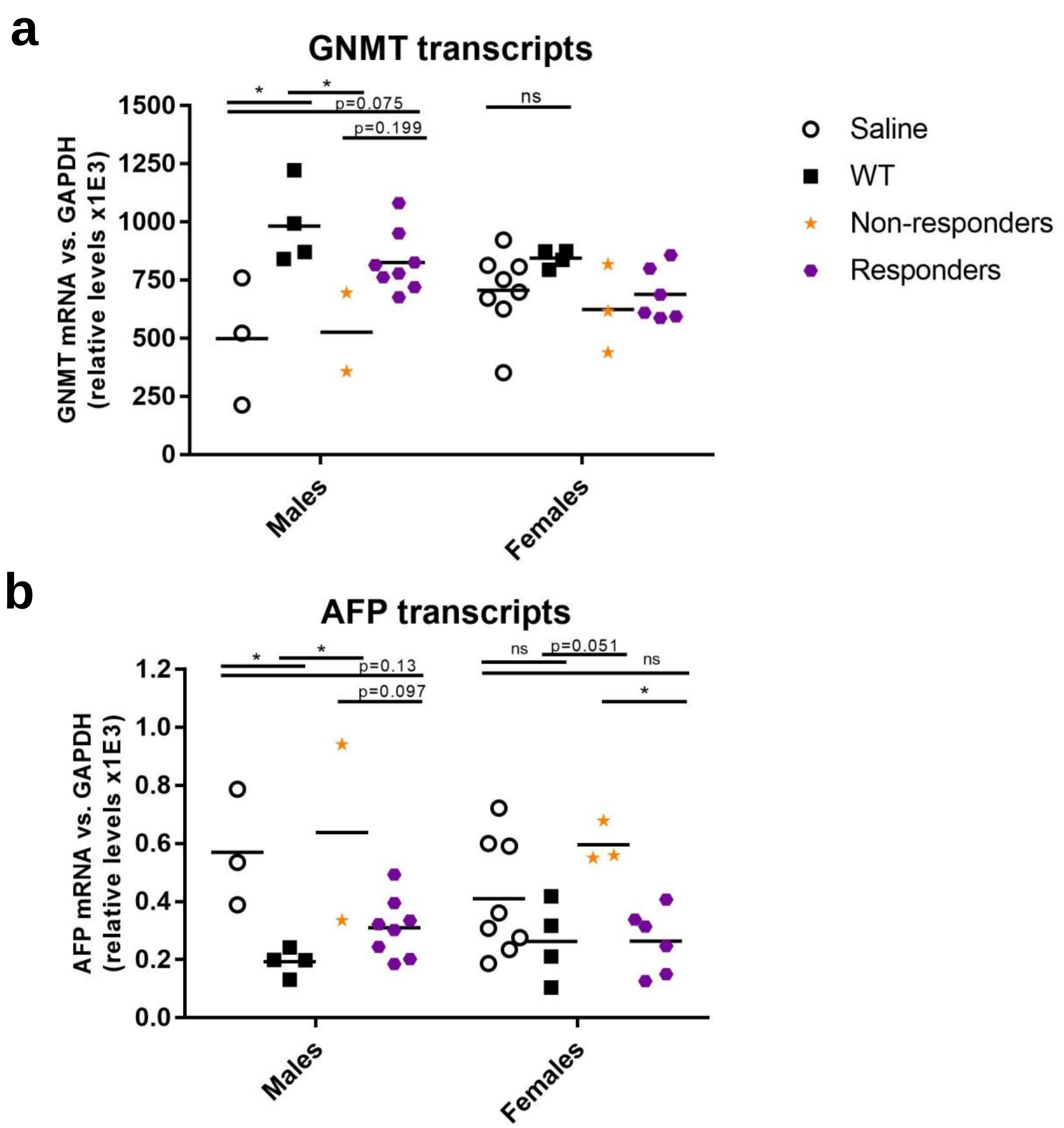

Supplementary Figure 4. Expression levels of the preneoplasia markers glycine N-methyltransferase (GNMT) and alpha fetoprotein (AFP) in *Abcb4*$^{-/-}$ mice treated with AAV-MDR3-Aco. Levels of GNMT (**a**) or AFP (**b**) transcripts were analysed by qRT-PCR relative to GAPDH transcripts and are shown for *Abcb4*$^{-/-}$ mice receiving treatment with AAV-MDR3 that achieved bile PC levels above 3900 μM at sacrifice (responders, purple hexagons; n=8M/6F) or did not achieve this threshold (non-responders, orange stars; n=2M/3F), along with saline-treated controls (open circles; n=3M/8F) and untreated WT mice (black squares; n=4M/4F). Statistics (one-way ANOVA/Tukey's multiple comparisons test): *, $p<0.05$; F values and degrees of freedom (numerator, denominator): (**a**) males: $F(3,13) = 5.671$, females: $F(3,17) = 1.591$; (**b**) males: $F(3,13) = 5.276$, females: $F(3,17) = 3.928$. Source data are provided as a Source Data file.

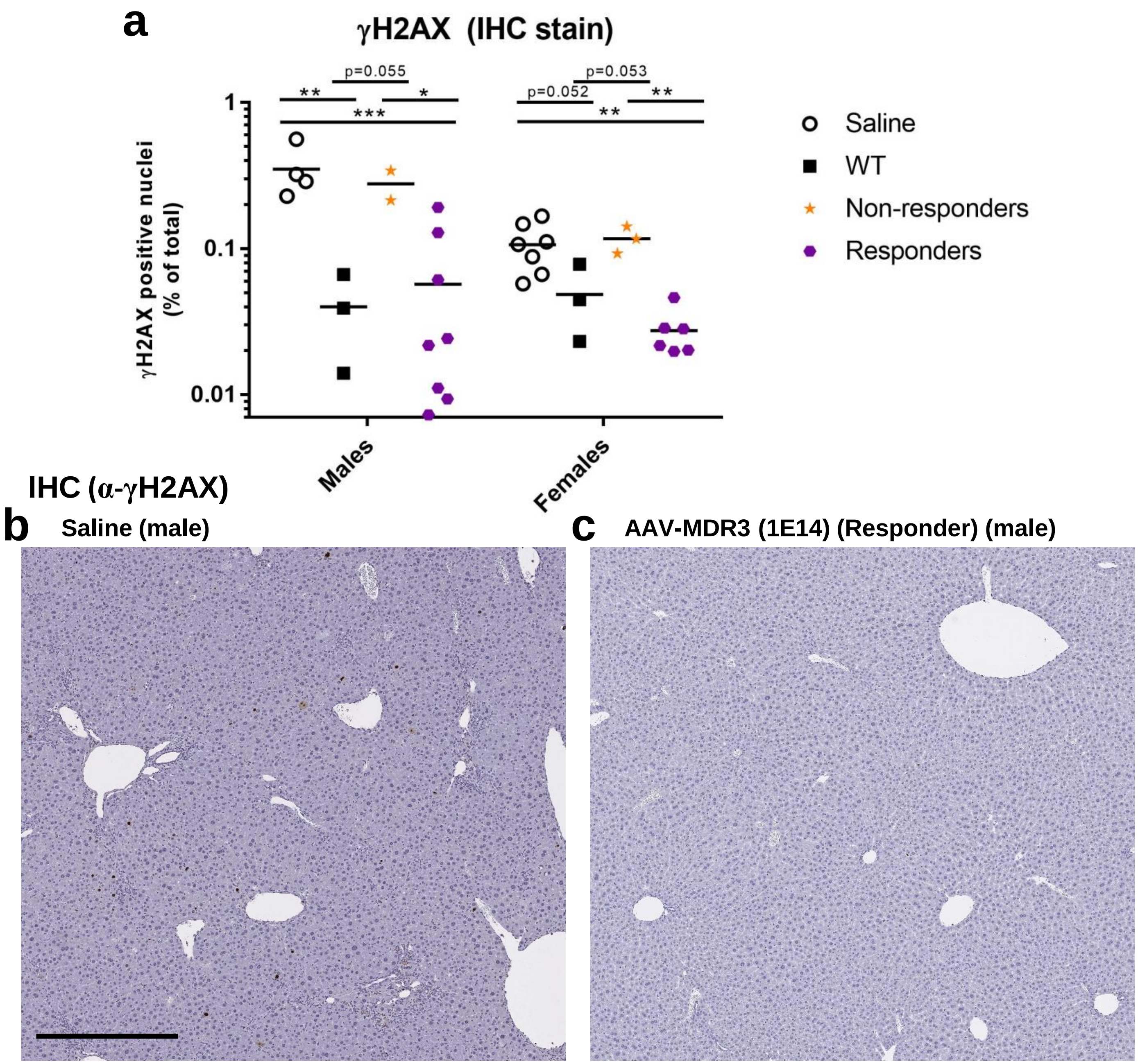

Supplementary Figure 5. Expression levels of the preneoplasia marker phosphorylated histone H2AX (γH2AX) in *Abcb4*$^{-/-}$ mice treated with AAV-MDR3-Aco. (**a**) Percentages of γH2AX-positive nuclei were analysed by IHC and are shown for *Abcb4*$^{-/-}$ mice receiving treatment with AAV-MDR3 that achieved bile PC levels above 3900 μM at sacrifice (responders, purple hexagons; n=8M/6F) or did not achieve this threshold (non-responders, orange stars; n=2M/3F), along with saline-treated controls (open circles; n=3M/8F) and untreated WT mice (black squares; n=4M/4F). Statistics (one-way ANOVA/Tukey's multiple comparisons test): *, $p<0.05$, **, $p<0.01$, ***, $p<0.001$; F values and degrees of freedom (numerator, denominator): males: $F(3,13) = 5.276$, females: $F(3,17) = 3.928$. (**b & c**) Representative images of IHC staining of γH2AX in nuclei of liver tissue of a saline-treated *Abcb4*$^{-/-}$ male (**b**) and AAV-MDR3-treated responder *Abcb4*$^{-/-}$ male (**c**). Scale bar = 500 μm. Source data are provided as a Source Data file.

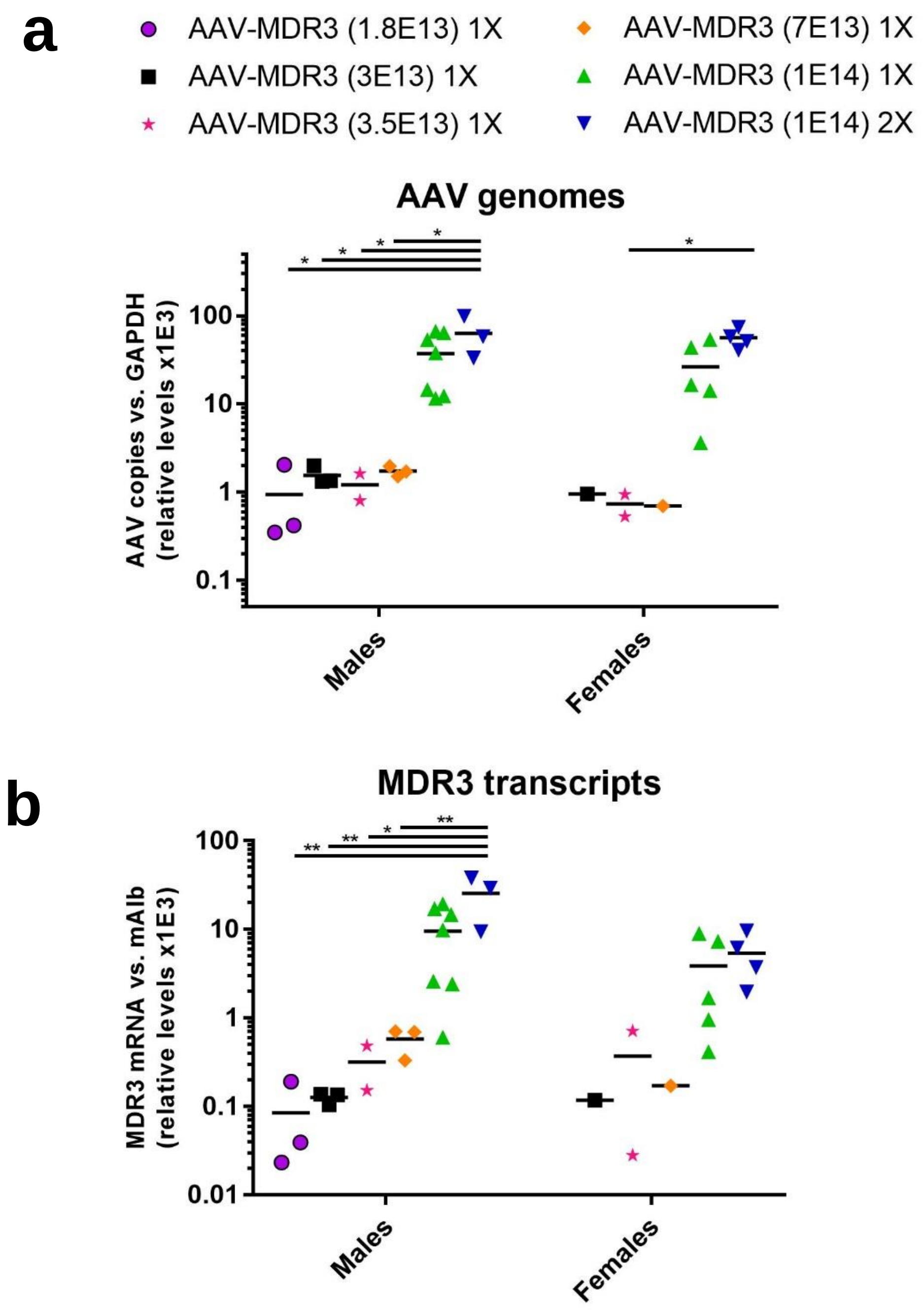

Supplementary Figure 6. Levels of AAV transduction and transgene expression for *Abcb4*$^{-/-}$ mice treated with AAV-MDR3-Aco. The levels of AAV genomes (**a**) and MDR3 mRNA transcripts (**b**), were quantified from liver tissue harvested from *Abcb4*$^{-/-}$ mice treated with AAV-MDR3-Aco at 1.8×10^{13} VG/kg 1X (purple circles), at 3×10^{13} VG/kg 1X (black squares), at 3.5×10^{13} VG/kg 1X (pink stars), at 7×10^{13} VG/kg 1X (orange diamonds) at 1×10^{14} VG/kg 1X (green triangles), or at 1×10^{14} VG/kg 2X (blue inverted triangles). AAV genomes and MDR3 transcripts were quantified via qPCR and RT-qPCR, respectively. Animals were sacrificed between 12 and 16 weeks after treatment. Statistics (one-way ANOVA/Tukey's multiple comparisons test): *, $p<0.05$; **, $p<0.01$; F values and degrees of freedom (numerator, denominator): (**a**) males: $F(5,15) = 5.88$, females: $F(4,8) = 5.145$; (**b**) males: $F(5,15) = 6.002$, females: $F(4,8) = 1.16$. Source data are provided as a Source Data file.

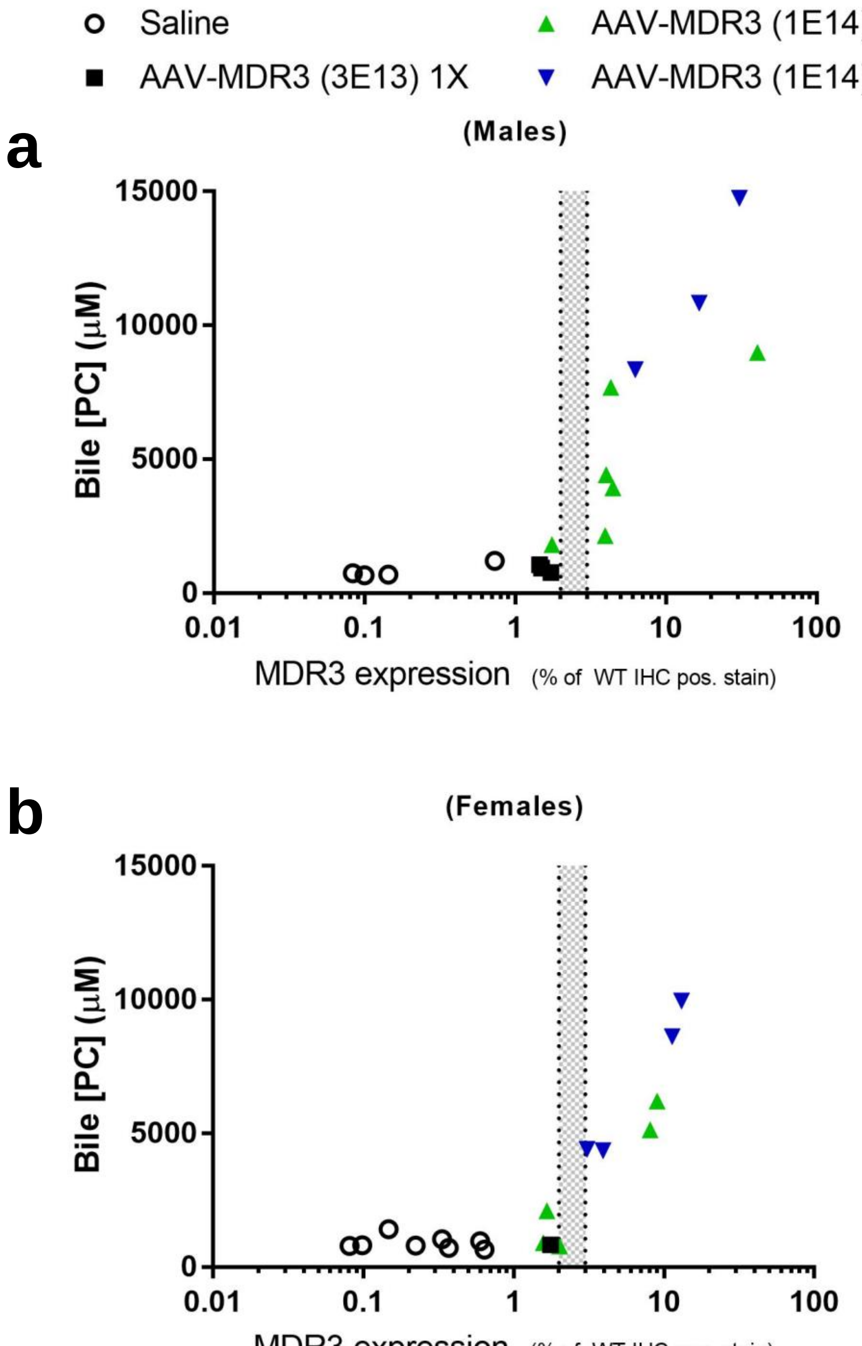

Supplementary Figure 7. MDR3 expression effect on bile PC levels in *Abcb4*$^{-/-}$ mice treated with AAV-MDR3-Aco. The PC concentration in the bile was plotted in relation to the MDR3 protein expression as quantified by the percent area of tissue that stained positive for MDR3 expression and normalized to levels in WT mice for male (**a**) and female (**b**) *Abcb4*$^{-/-}$ mice treated with AAV-MDR3-Aco at 3x10^{13} VG/kg 1X (black squares), at 1x10^{14} VG/kg 1X (green triangles), or at 1x10^{14} VG/kg 2X (blue inverted triangles), as well as saline-treated controls (open circles). The vertical shadowed stripe indicates the threshold of MDR3 expression above which AAV-MDR3 treatment is therapeutic. Source data are provided as a Source Data file.

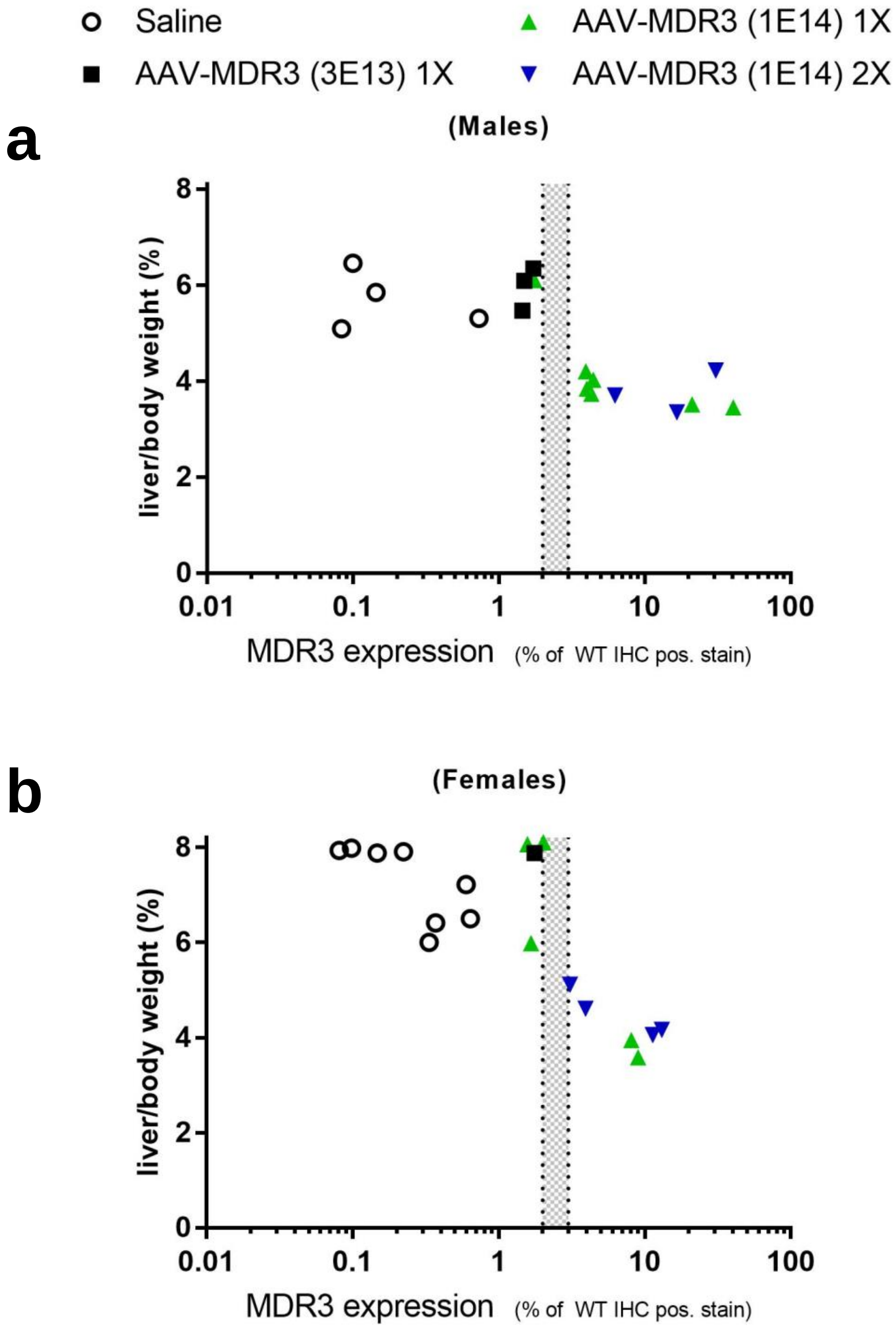

Supplementary Figure 8. MDR3 expression effect on liver weight in *Abcb4*$^{-/-}$ mice treated with AAV-MDR3-Aco. The liver weight as a percent of body weight was plotted in relation to the MDR3 protein expression as quantified by the percent area of tissue that stained positive for MDR3 expression and normalized to levels in WT mice for male (**a**) and female (**b**) *Abcb4*$^{-/-}$ mice treated with AAV-MDR3-Aco at 3x10^{13} VG/kg 1X (black squares), at 1x10^{14} VG/kg 1X (green triangles), or at 1x10^{14} VG/kg 2X (blue inverted triangles), as well as saline-treated controls (open circles). The vertical shadowed stripe indicates the threshold of MDR3 expression above which AAV-MDR3 treatment is therapeutic. Source data are provided as a Source Data file.

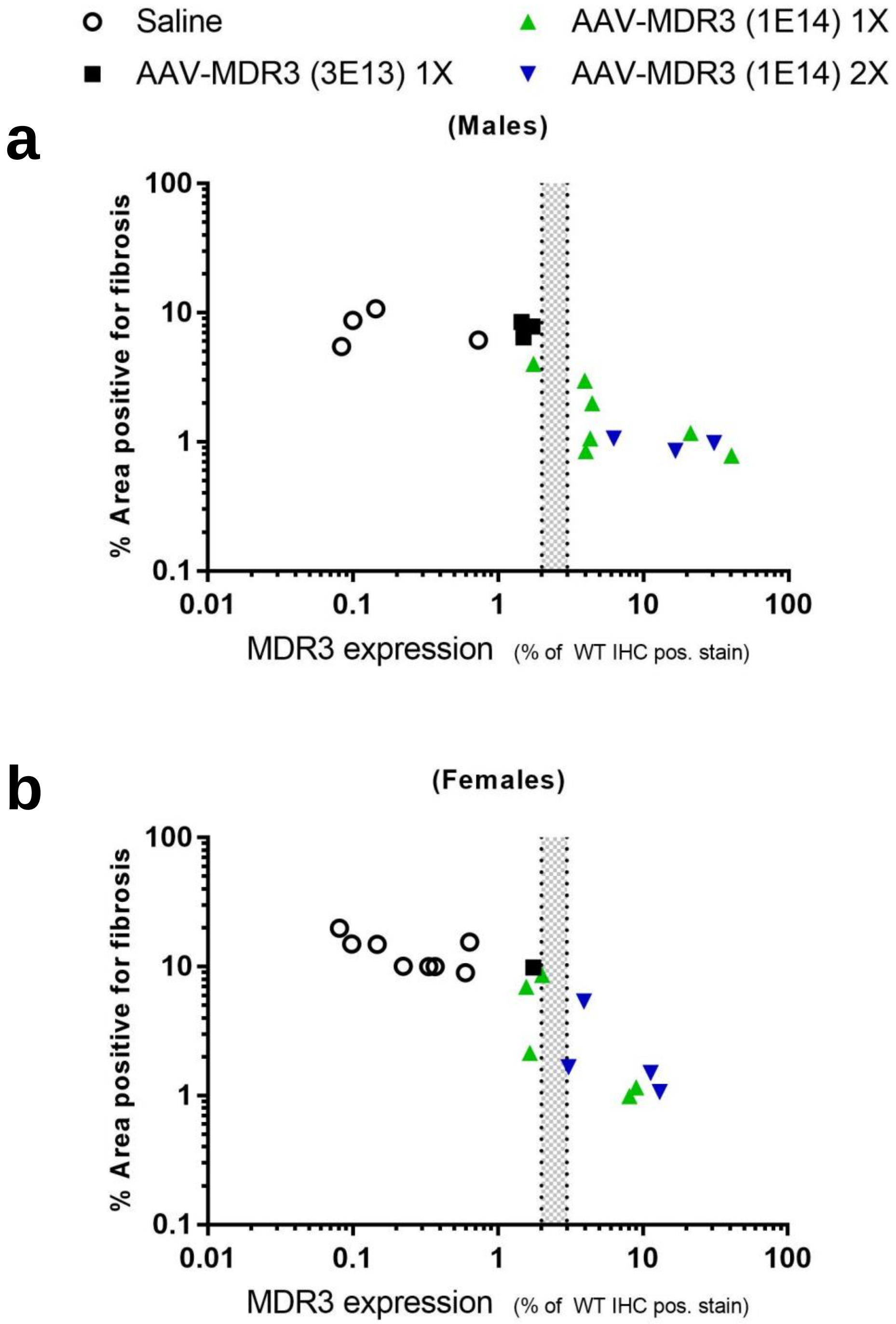

Supplementary Figure 9. MDR3 expression effect on fibrosis in *Abcb4*$^{-/-}$ mice treated with AAV-MDR3-Aco. The extent of liver fibrosis detected via picrosirius red staining was plotted in relation to the MDR3 protein expression as quantified by the percent area of tissue that stained positive for MDR3 expression and normalized to levels in WT mice for male (**a**) and female (**b**) *Abcb4*$^{-/-}$ mice treated with AAV-MDR3-Aco at 3x10^{13} VG/kg 1X (black squares), at 1x10^{14} VG/kg 1X (green triangles), or at 1x10^{14} VG/kg 2X (blue inverted triangles), as well as saline-treated controls (open black circles). The vertical shadowed stripe indicates the threshold of MDR3 expression above which AAV-MDR3 treatment is therapeutic. Source data are provided as a Source Data file.

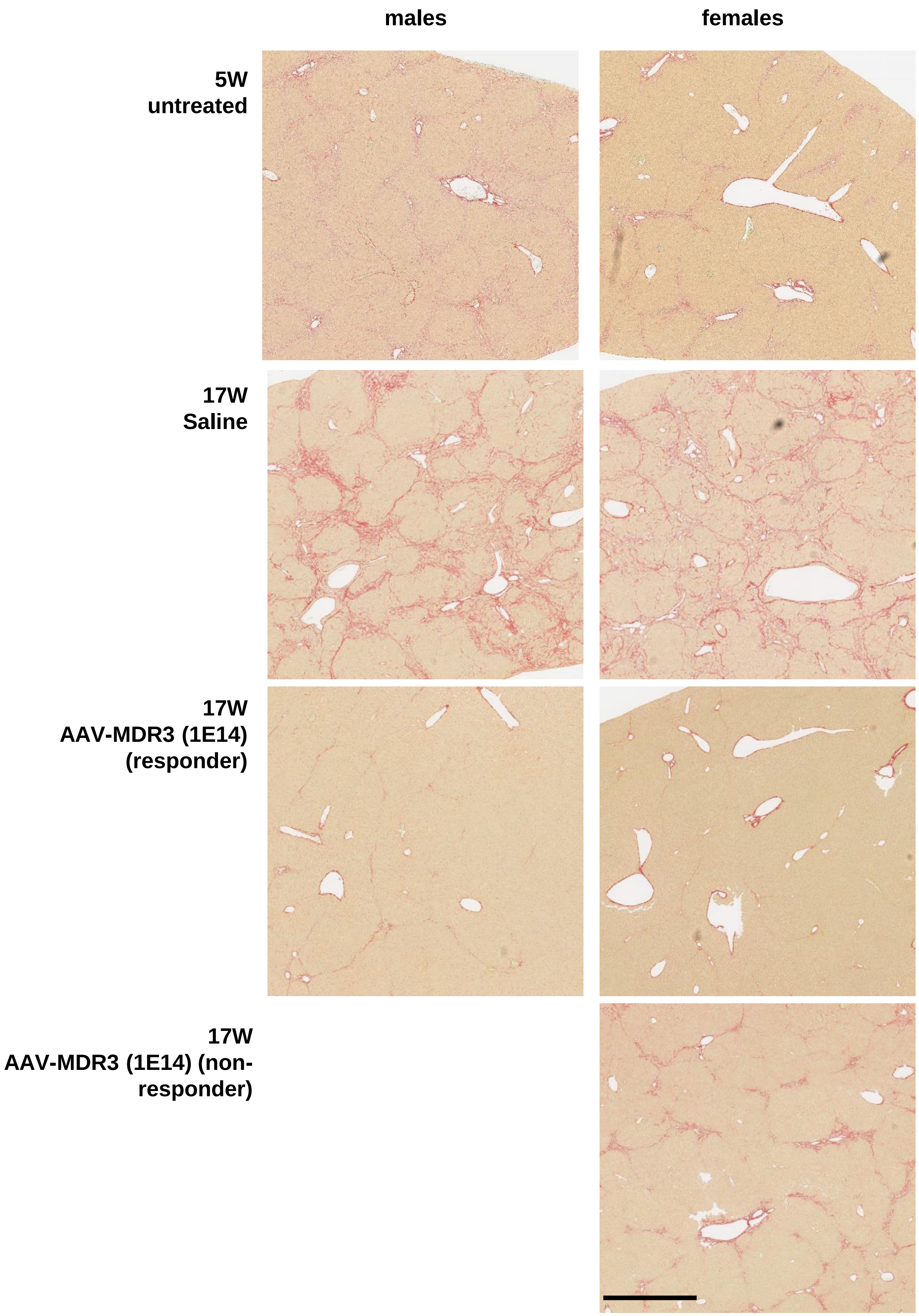
males
females
5W
untreated
17W
Saline
17W
AAV-MDR3 (1E14)
(responder)
17W
AAV-MDR3 (1E14) (non-
responder)

Supplementary Figure 10. Fibrosis in *Abcb4*$^{-/-}$ mice treated at five weeks of age with AAV-MDR3-Aco. Representative images of picrosirius red staining of mouse liver sections for five-week-old mice at therapy initiation and 12 weeks post treatment for mice treated with saline or AAV-MDR3 (1E14) 1X showing males on the left and females on the right. Scale bar = 1mm.

Supplementary Table 1. Primer list

Oligonucleotide name	Sequence (5´-3´)
A1AT-Pr_F	TTGCTCCTCCGATAACTGGG
A1AT-Pr_R	CCCTGTCCTCGTCCGTATTT
GAPDH_F	GGATGCAGGGATGATGTTC
GAPDH_R	TGCACCACCAACTGCTTA
GNMT_F	GTGTGTGGCAGCTGTACATCG
GNMT_R	CAAAGCCATCTCCTGAAAGCAC
AFP_F	AAAATTTGGATCCCGAAACC
AFP_R	TGCGTGAATTATGCAGAAGC
