# Supplementary material for: Gene therapy for progressive familial intrahepatic cholestasis type 3 in a clinically relevant mouse model
Source: Nat Commun. 2019 Dec 13;10:5694. doi: 10.1038/s41467-019-13614-3 (PMC6910969; doi:10.1038/s41467-019-13614-3)
Supplement: Supplementary file 3 — Reporting Summary [file 41467_2019_13614_MOESM3_ESM.pdf]

nature research

Corresponding author(s): Nicholas D Weber
Gloria González-Aseguinolaza
Cristian Smerdou

Last updated by author(s): Sep 25, 2019

Reporting Summary

Nature Research wishes to improve the reproducibility of the work that we publish. This form provides structure for consistency and transparency in reporting. For further information on Nature Research policies, see Authors & Referees and the Editorial Policy Checklist.

Statistics

For all statistical analyses, confirm that the following items are present in the figure legend, table legend, main text, or Methods section.

n/a	Confirmed	
☐	☒	The exact sample size (n) for each experimental group/condition, given as a discrete number and unit of measurement
☐	☒	A statement on whether measurements were taken from distinct samples or whether the same sample was measured repeatedly
☐	☒	The statistical test(s) used AND whether they are one- or two-sided<br>*Only common tests should be described solely by name; describe more complex techniques in the Methods section.*
☒	☐	A description of all covariates tested
☒	☐	A description of any assumptions or corrections, such as tests of normality and adjustment for multiple comparisons
☐	☒	A full description of the statistical parameters including central tendency (e.g. means) or other basic estimates (e.g. regression coefficient) AND variation (e.g. standard deviation) or associated estimates of uncertainty (e.g. confidence intervals)
☒	☐	For null hypothesis testing, the test statistic (e.g. F, t, r) with confidence intervals, effect sizes, degrees of freedom and P value noted<br>*Give P values as exact values whenever suitable.*
☒	☐	For Bayesian analysis, information on the choice of priors and Markov chain Monte Carlo settings
☒	☐	For hierarchical and complex designs, identification of the appropriate level for tests and full reporting of outcomes
☒	☐	Estimates of effect sizes (e.g. Cohen's d, Pearson's r), indicating how they were calculated

Our web collection on statistics for biologists contains articles on many of the points above.

Software and code

Policy information about availability of computer code

Data collection	Immunohistochemistry and histology images were collected using Aperio Scanner Console (version 102.0.7.5) or ZEN 2.3 (blue edition)<br>Immunofluorescence and confocal images were collected using ZEN 2.3 (blue edition)
Data analysis	Software for graphs:<br>- Graphpad PRISM version 7.05<br><br>Software for image processing:<br>- ImageJ version 1.48v<br>- Custom ImageJ macros for collagen, MDR3, and γH2AX expression detection (code is available upon request)<br>- Aperio ImageScope version 12.1.0.5029<br><br>Other software:<br>- Microsoft PowerPoint version 1812

For manuscripts utilizing custom algorithms or software that are central to the research but not yet described in published literature, software must be made available to editors/reviewers. We strongly encourage code deposition in a community repository (e.g. GitHub). See the Nature Research guidelines for submitting code & software for further information.

Data

Policy information about availability of data

All manuscripts must include a data availability statement. This statement should provide the following information, where applicable:

- Accession codes, unique identifiers, or web links for publicly available datasets
- A list of figures that have associated raw data
- A description of any restrictions on data availability

The authors declare that the all the data supporting the findings of this study are available within the article and the Supplementary Information files.

Field-specific reporting

Please select the one below that is the best fit for your research. If you are not sure, read the appropriate sections before making your selection.

☒ Life sciences ☐ Behavioural & social sciences ☐ Ecological, evolutionary & environmental sciences

For a reference copy of the document with all sections, see nature.com/documents/nr-reporting-summary-flat.pdf

Life sciences study design

All studies must disclose on these points even when the disclosure is negative.

Sample size	Sample sizes were chosen based on prior experience and quantity of test article available per study. No statistical predetermination of sample size was done.
Data exclusions	- One mouse from each of 2X treatment groups (2-week old males and females groups) was excluded due to very low transgene expression indicating that it had not been properly inoculated with the test article.<br>- One mouse from each of 2X treatment groups and one female from the 1X five-week old treatement group did not provide terminal data due to death for unknown causes prior to study completion. Serum biomarker data for these mice were still obtained and included in the analysis.
Replication	Study data were compiled from three repetitions of the same experimental outline. Study-to-study variation was not observed.<br>Phosphatidylcholine samples were assayed in duplicate.<br>Samples analyzed by qPCR were assayed in triplicate.
Randomization	Mice were randomly allocated to experimental groups.
Blinding	The investigators were not blinded to group allocation during data collection and/or analysis.

Reporting for specific materials, systems and methods

We require information from authors about some types of materials, experimental systems and methods used in many studies. Here, indicate whether each material, system or method listed is relevant to your study. If you are not sure if a list item applies to your research, read the appropriate section before selecting a response.

Materials & experimental systems

n/a	Involved in the study	
☐	☒	Antibodies
☐	☒	Eukaryotic cell lines
☒	☐	Palaeontology
☐	☒	Animals and other organisms
☒	☐	Human research participants
☒	☐	Clinical data

Methods

n/a	Involved in the study	
☒	☐	ChIP-seq
☒	☐	Flow cytometry
☒	☐	MRI-based neuroimaging

Antibodies

Antibodies used	For Immunofluorescence:<br>- Anti-MDR3 (Millipore clone P3-II-26. ref. MAB4140)<br>- Donkey anti-mouse IgG Alexa-488-conjugated antiserum (Invitrogen, ref. A21202)<br>For Immunohistochemistry (IHC):<br>- Anti-ABCB4/MDR3 Antibody (IHC-Plus) (LS-Bio, ref. LS B5729).<br>- Anti-phosphorylated histone H2AX (γH2AX) (#9718, Cell Signalling, Danvers MA)<br>- Goat anti-rabbit EnVision (Dako, ref. K4002)

Validation	Commercially available antibodies were validated by the vendors.<br>In addition, anti-ABCB4/MDR3 Antibody (IHC-Plus) was validated by IHC using different dilutions in liver samples of FVB.Mdr2+/+ (Abcb4+/+) mice using as negative control liver samples from FVB.Mdr2−/− mice (Abcb4−/−)

Eukaryotic cell lines

Policy information about cell lines

Cell line source(s)	HuH-7: Japanese Collection of Research Bioresources Cell Bank: 0403<br>HEK-293T: ATCC CRL-3216
Authentication	Cell line authentication was originally performed by the vendor/Cell Bank.
Mycoplasma contamination	Cell lines tested negative for mycoplasma contamination.
Commonly misidentified lines<br>(See ICLAC register)	*Name any commonly misidentified cell lines used in the study and provide a rationale for their use.*

Animals and other organisms

Policy information about studies involving animals; ARRIVE guidelines recommended for reporting animal research

Laboratory animals	We used 2- and 5-week-old male and female FVB.Mdr2+/+ (Abcb4+/+) mice from Envigo and in-house bred homozygous FVB.Mdr2-/- (FVB.129P2-Abcb4<tm1Bor>/J) male and female mice.
Wild animals	This study did not involve wild animals.
Field-collected samples	This study did not involve samples collected from the field.
Ethics oversight	All animal experiments and procedures were reviewed and approved by the Universidad de Navarra Institutional Ethical Committee (protocol numbers: 082c-17 for breeding and 086-17 for animal studies).

Note that full information on the approval of the study protocol must also be provided in the manuscript.
